# Unraveling interlanguage variability: A psycholinguistic exploration of morphological forms in Iranian ESL learners' written performance

**DOI:** 10.1016/j.heliyon.2024.e31582

**Published:** 2024-05-24

**Authors:** Forough Amirjalili, Masoud Neysani, Ahmadreza Nikbakht

**Affiliations:** Department of English Language and Literature, Yazd University, Yazd, Iran

**Keywords:** Cognitive processes, ESL learners, Interlanguage variability, Morphological forms, Psycholinguistic aspects

## Abstract

The present research study aimed to investigate the psycholinguistic aspects of interlanguage (IL) variability in English as a second language learners from Iran. Three morphological forms were specifically examined in this study: the plural "-s," past tense forms, and present tense forms. In order to investigate the learners' cognitive processes, the researchers used a mixed-method approach, combining qualitative semi-structured interviews with quantitative analysis of written task performance. Under various task conditions, learners exhibited two opposing patterns of IL variability (increasing and decreasing) in their morphological forms. Various degrees of accuracy were noted for every kind of morpheme. Different students allocated their attention differently to form and meaning in both under pressure and relaxed task environments. The observed variations could have been caused by limited working memory and attention capacity in addition to L1 transfer. The research indicated that the field of second language acquisition could benefit from an explanatory approach to comprehend IL variability from the learner's perspective. It provides recommendations for larger-scale research projects in a variety of educational contexts, along with pedagogical implications.

## 1 Introduction

Throughout the development of a second language (L2), phrases like "Yesterday he is sick" and "Last year we visited my friend" are examples of "non-target-like" expressions produced by English language learners [1] Examples include "My brother works in Portland but lives in San Francisco" [2] and verb form variations like "walked" and " talked." Tarone [3] has underscored the intermittent correctness and incorrectness of morphological rules related to negation and verb tense. The term IL refers to this unique language, which is different from the target language as well as the learner's native tongue, like English [[4], [5], [6],^7^].

IL is characterized by variability [8,9], where learners exhibit fluctuations in their usage of correct and incorrect forms. This variability is considered a prominent feature of learner interlanguage and is believed to significantly impact language development [10]. Expanding the scope, IL variation is observed when learners produce language in diverse contexts, and researchers have explored it from linguistic [11,12], psycholinguistic [13,8], and sociolinguistic [14] perspectives.

From a psycholinguistic standpoint, scholars contend that IL variability arises as learners engage in tasks under different 'planning conditions' [8,15,16]. This perspective suggests a triadic relationship involving variability, planning, and psycholinguistic processes in the production of L2 learners' IL. An increasing amount of psycholinguistic research such as Kellogg's [15] model of writing and Skehan's [16] hypothesis of Limited Attention Capacity suggests that learners who encounter different task planning conditions will show variability in IL. Pre-task planning, or activities done prior to a task, and within-task planning, or online planning done while performing a task, are two broad categories into which these conditions fall. According to within-task planning conditions, the latter category of time conditions is further subdivided into unpressured (unlimited time) and pressured (limited time) situations [8].

One common claim [1‍2] in the context of within-task planning conditions is that learners tend to perform tasks under time-unpressured conditions with greater accuracy in grammatical forms than they do under time-pressured conditions. Differences in the cognitive processes of the learners under the two planning scenarios are thought to be the cause of this discrepancy. This hypothesis is theoretically supported by the idea that working memory and attention capacity constraints under pressure cause learners to devote greater focus on meaning (lexicalized knowledge) as opposed to form (rule-based knowledge) [15,17,16,18]. Consequently, learners can use the extra time to retrieve resources from their long-term memory for vocabulary and grammar when given more time to plan their tasks [8,19].

In contrast to pre-task planning, which has received more research attention [20,21,22,23,24,25,26], there has been comparatively less focus on within-task planning conditions. While research over the past decades has explored interlanguage (IL) variability of grammatical morphemes, the findings have been inconsistent. Studies by Refs. [27,28] have found a significant difference in task performance when time conditions change, while studies by Refs. [29,30] have not found any such difference. Therefore, additional study is required to fully comprehend how various within-task planning circumstances affect IL variations in grammatical morphemes.

As proposed by Ref. [4], the "five central psycholinguistic processes" may also play a role in explaining IL variability, in addition to working memory and attention capacity. Research studies from the past and present have consistently found that learners' linguistic performance during L2 development is influenced by both L1 transfer and training transfer, in particular [31,32,3]. There is evidence that factors such as L1 transfer may contribute to IL variation, despite the fact that one line of research highlights working memory and attention capacity as the primary limitations on the explanation of IL variability in within-task planning scenarios [33,34]. Notably, despite the exploration of various L1 backgrounds, there is a gap in the literature concerning Iranian learners of English, making a targeted study on this homogeneous group a noteworthy endeavor.

Despite the existing research that delves into the influence of factors such as working memory, attention capacity, and other cognitive constraints like L1 transfer IL variability, Studies that collect information on learners' perspectives in order to obtain a deeper understanding of their true thought processes are conspicuously rare [27,35]. Crucially, as students advance through different language proficiency levels, comprehending their reflections becomes essential, offering insightful information about individual variations in performance and thought processes [36,37]. In order to fill this gap, the current study used a psycholinguistic lens to investigate IL variability of three morpheme types in the case of Iranian ESL students.

The three morphemes that are being targeted are the plural "-s," past and present tense forms. These three morpheme types were chosen for several reasons.

1. **Commonly Used**: Past tense forms, present tense forms, and plural forms are among the most commonly used morphemes in English grammar. They are foundational elements of constructing sentences in English, making them crucial for language learners to grasp.
2. **Error-Prone Areas**: These morphemes are often error-prone for language learners, especially those whose native language differs significantly from English in terms of verb conjugation and pluralization rules. Identifying and addressing errors in these areas can lead to significant improvements in overall language proficiency.
3. **L1 Transfer**: Persian, the native language of the Iranian participants in the study, lacks inflectional morphemes for tense and pluralization. This structural difference between Persian and English makes these morphemes particularly susceptible to L1 transfer errors. By focusing on these morphemes, the study aims to investigate the extent to which L1 transfer influences learners' interlanguage.
4. **Literature Support**: Previous studies have shown that errors in past and present tense forms, as well as pluralization, are commonly attributed to L1 transfer among learners of different L1 backgrounds, including Iranian learners of English. By targeting these specific morphemes, the study contributes to the existing literature on IL variability and the role of L1 transfer in second language acquisition.

In particular, the study looked into how learners under pressured and unpressured within-task planning circumstances utilized these morphological forms differently, leading to IL variability in written tasks. Furthermore, through one-on-one retrospective interviews, the researchers aimed to identify the cognitive processes that learners invoked during these tasks.

In various respects, this study advanced the field of second language acquisition. First off, by examining IL variability in morphological forms under within-task planning conditions as an area with few reported studies. Secondly, it added to the body of literature. Thirdly, it focused on Iranian ESL learners, a group that has been relatively understudied. Fourthly, in addressing sources of IL variability, the study considered various cognitive constraints, including the potential impact of negative L1 transfer. Fifthly, it provided additional evidence for understanding IL variability by incorporating learners' retrospection, aligning with suggestions from Ref. [2] on the value of verbal self-reports. Finally, the study informed researchers about an approach to examining IL variability. As a result of this, the present investigation tried to find a suitable answer to the following research questions.

RQ1 Under both pressured and unpressured within-task planning conditions, how much do learners exhibit patterns of IL variability in the targeted morphological forms?

RQ2 How much do students display varying degrees of accuracy in these morphological forms?

RQ3 How do learners' attentional resources change under pressured and unpressured within-task planning conditions?

RQ4 How much does IL variability involve L1 transfer and training transfer?

## 2 Literature review

### 2.1 A historical overview of IL theory

The study of learner language has been a focal point in second language acquisition, prompting the development of prominent theories in the latter half of the twentieth century. When [38] first put forth the Contrastive Analysis Hypothesis (CAH), the goal was to compare the learner's native language system with the target language system in order to analyze learner's language. While CAH predicted learning difficulties based on language differences, its limitations became evident, leading to the emergence of Error Analysis (EA) by Ref. [39]. EA focused on analyzing samples of learner language, moving away from the exclusive attribution of errors to native language interference. However, EA also faced criticism, paving the way for the Interlanguage Theory introduced by Selinker [4].

The term "interlanguage," which was first used by Ref. [4] in 1972, refers to a linguistic system that second language (L2) learners have developed that is separate from both their target language and their native tongue. Also referred to as the 'approximative system' and 'idiosyncratic dialect,' interlanguage is observable as an 'in-between' language, demonstrating both target-like and non-target-like forms. This phenomenon establishes the foundation for IL variability, encapsulated in 'interim grammars or mental representations of grammar persisting in learners' competence.

IL variability is a well-explored characteristic denoting the instability of learner language during L2 production. It is observed as the use of variable forms, with correct and incorrect forms coexisting at different stages of L2 development. This variability can be systematic or non-systematic, with the former exhibiting consistent patterns under different conditions [27]. While systematic variability is acknowledged widely, non-systematic or free variation exists where linguistic forms are chosen independently of psycholinguistic or sociolinguistic contexts [3].

Research on IL variability mainly has evolved with three main approaches; linguistic, sociolinguistic, and psycholinguistic. The linguistic approach emphasizes learners' performance, relying on the homogeneous competence paradigm and learners' introspection. Sociolinguistic perspectives, following the Labovian paradigm, consider style shifting as central to L2 variability. The psycholinguistic approach examines IL variability through learners' mental processing, utilizing planning and monitoring models [33].

### 2.2 IL variability: a psycholinguistic perspective

#### 2.2.1 Working memory and attention capacity

Interlanguage (IL) variability is largely explained by working memory and attention, especially when task planning is involved. The trade-off effect is introduced by Kellogg's [15] writing model, which contends that time constraints during written tasks determine one's capacity for monitoring—that is, the ability to correct linguistic errors before or after execution. Skehan [16] contributes the Limited Attention Capacity hypothesis, linking learners' cognitive processes to language aspects (fluency, accuracy, complexity) under varied planning conditions. This is consistent with the theory that learners prioritize meaning over form or lexicalized knowledge over rule-based knowledge because they have limited working memory and attention [17,18].

#### 2.2.2 L1 transfer and transfer of training: possible 'co-sources'

L1 transfer and transfer of training, derived from Selinker's [4] psycholinguistic processes, are key factors influencing IL variability. While training transfer results from using rules acquired from materials or instructions, L1 transfer is the impact of the native language on the target language. These cognitive processes, along with working memory and attention, are considered 'co-sources' contributing to IL variations. Studies by Refs. [31,32,3] highlight the combined influence of these cognitive factors on language learner performance and highlight their function as limitations.

### 2.3 Task-based planning: focus on within-task planning conditions

Task planning conditions, a psycholinguistic perspective, are crucial for understanding IL variability. Ellis's [2] framework classifies planning into pre-task and within-task, the latter further divided into pressured and unpressured conditions. Pressured planning involves completing tasks rapidly with limited time, while unpressured planning allows learners unlimited time [2,19]. support theoretical claims that more time in unpressured conditions enables learners to allocate attention to linguistic forms, retrieve rule-based knowledge, and monitor effectively, leading to increased accuracy in language use. The present study focuses on within-task planning due to its limited exploration compared to pre-task planning. This is because, as opposed to pre-task planning [20,21,22,23,24,25,26], very few studies have investigated IL variability under pressured and unpressured within-task planning conditions.

### 2.4 Research on IL variability under within-task planning

The complex relationship between learners' cognitive processes, interlanguage (IL) variability, and within-task planning conditions has been the subject of research in recent years. Two distinct groups of studies present divergent conclusions.

Group 1: No Difference in Performance

According to the first set of research, there is no discernible difference in learner performance under pressured and unpressured within-task planning circumstances [35]. experiment on story retelling, focusing on subject-verb inversion and finite verb position in Dutch learners, found that time pressure had minimal impact compared to the focus on grammar. Similarly, Caudery [29] explored writing tasks and observed no score differences between timed and untimed conditions. Wang's [30] study on Chinese EFL learners' language use in storytelling under different planning conditions indicated limited effects on speech accuracy, with online planning showing minimal impact.

Group 2: Strong Connection Between Planning and Performance

In contrast, there is strong evidence from a different set of studies linking language performance, cognitive processes, and within-task planning. Ellis [28] showed that learners with mixed backgrounds could produce morphemes with systematically varying accuracy, with accuracy decreasing as planning opportunities decreased due to time constraints. Yuan and Ellis [34] focused on accuracy, complexity, and fluency in a picture-based story-writing task for Chinese EFL learners. The study revealed higher rates of accurate grammar use in unpressured than pressured conditions, emphasizing the impact of planning time on linguistic form accuracy. Participants tended to allocate attention to lexico-grammatical resources when given unlimited time. These studies contribute valuable insights into the nuanced relationship between planning conditions, cognitive processes, and IL variability, shedding light on the complexity of language production under different task contexts.

### 2.5 Joint effects of cognitive factors on IL variability

In Long's [33] case study, the impact of time conditions on a learner's performance in both descriptive speaking and untimed essay writing tasks was explored. Notably, the learner exhibited differences in past tense form accuracy between the spoken and written tasks, attributed to a potential trade-off effect. Under time constraints, the learner focused more on meaning than form in the spoken task. Moreover, L1 transfer and training transfer were found to be factors in IL variability in linguistic forms. However, the study raised two unresolved issues: the absence of information on pre-task planning time for the speaking task and limited clarification on the connection between interviews and task performance.

Abdi Tabari [27] conducted a more recent study in which IL variability was examined in relation to different planning scenarios, such as no planning, pre-task planning, pre-task/pressured online planning, and pre-task/pressured online planning. Participants had to complete written assignments that required them to tell stories using pictures. According to the study, when compared to the no planning group, the pressured online planning group produced language more accurately, including using the right verb forms. This was explained by the students' propensity to focus more of their online planning time on looking up grammatical resources. While the study incorporated learners' retrospection, it involved only a subset of participants, potentially limiting generalizability.

There is a lack of research on Iranian English language learners in this context, despite the fact that some studies [28,35,33] have examined IL variability in within-task planning conditions for grammatical morphemes. Nguyen and Newton [25] looked into morphemes, specifically the copula 'be' and the third-person singular '-s' and concluded that negative L1 transfer and limited working memory capacity contributed to errors. However, this study employed pre-task planning, offering an opportunity for the present study to complement the focus on within-task planning conditions. Working memory and attention limitations have been the main focus of explanations for IL variability under task planning conditions in recent years. In order to fully explain learners' IL variability between task planning conditions, this study aims to broaden the scope by incorporating L1 transfer and transfer of training as potential "joint-sources" in addition to working memory and attention constraints.

In conclusion, the above-mentioned literature review highlights several gaps in the current research related to within-task planning conditions and their effects on learners' IL variability in written language performance. The following three main gaps can be identified.

1. Limited Studies on Within-Task Planning Conditions:

First Gap: Empirical studies [25] on IL variability in within-task planning conditions are fewer compared to those focusing on pre-task planning. Although the effects of pressured and unpressured within-task planning conditions have yielded inconsistent results, the majority of studies have focused on various aspects of language, including fluency, accuracy, and complexity, with few fully investigating IL variation in morphological forms. Thus, there is a need for further investigation specifically examining IL variability in morphological forms under within-task planning conditions.

2. Lack of Consistency in Task Type and Time Conditions:

Second Gap: Few research [8,40] has examined learner IL variability in both timed and untimed conditions while using the same written mode to avoid confusing spoken and written tasks. Mixing and matching task types within a single study design may result in confusing modality conditions and planning. Therefore, the present study aims to investigate whether learners exhibit similar patterns of IL variability under the same task type (written) when time is a variable.

3. Limited Consideration of Cognitive Processes:

Third Gap: The extant literature [27,33] frequently highlights the restricted ability of working memory and attention to account for interstitial lung variability, particularly in situations involving within-task planning. To account for IL variation, few studies have simultaneously examined working memory, attention span, and other cognitive processes, such as transfer of learning and L1 transfer. Furthermore, very few studies have used interviews conducted under timed and untimed conditions to try and glean insight into learners' thought processes.

## 3 Methodology

### 3.1 Participants

Iranian ESL students who study English language teaching at Yazd University participated in the present study. The exploration included a small-scale group of five participants, allowing for a qualitative component to gain deeper insights into individual cognitive processes during the written performance task. Participants were ESL learners with Persian as their first language (L1). The study intentionally avoided eliciting additional demographic information (e.g., age, ethnicity, study majors) to maintain participant anonymity and focus on psycholinguistic aspects.

Participants were contacted simultaneously through two different channels. The initial method utilized virtual social networks, specifically designed for connecting Iranian students at Yazd University, to post the advertisement on popular sites. The second method involved the researcher use their existing social network with Irani students to promote the study through email communication.

Additionally, a 'snowball sampling' strategy was employed through these two channels, whereby individuals who viewed the advertisement were encouraged to pass it along to others who may have had an interest. Despite the initial plan to recruit participants through physical meetings with potential participants in classrooms, this strategy was unnecessary as the desired number of participants was already achieved through the methods described above.

Prospective participants were encouraged to contact the researcher for additional information, as outlined in the advertising. The individuals who expressed interest were sent a follow-up email containing an electronic copy of the participant information and consent form. This allowed them to review the materials in advance and make an informed decision about whether or not to attend the research in person. Ultimately, a total of five subjects willingly consented to participate in the investigation. Table 1 shows the participants’ demographic information.

**Table 1:** Participants’ demographic data.

Groups	N	Age	Gender	Major	Percent (%)
ESL Students	5	18–30	Female	TEFL	100

### 3.2 Research design

The study focused on three morpheme types, each related to different tense forms. In fact, by using these three morpheme types, the research team wanted to investigate the interlanguage variability among Iranian learners of English. These three types of morphemes are presented as follow.

a) Past Tense Forms:

Regular Past Tense Form:

Targeted morpheme: '-ed'

Irregular Past Tense Form:

This includes using different verbs in the main and auxiliary constructions of the past perfect and simple past tenses (both active and passive voice).

b) Present Tense Forms:

Regular Third Singular Person 's' (3SG-s):

Targeted morpheme: '-s'

Irregular Present Tense Form:

This included using different verbs in the main and auxiliary constructions of the present perfect and simple present tenses (both active and passive voice).

Note on Error Counting:

In instances where a different tense was used than required by the context, it was considered incorrect. When a student wrote, "I go to school yesterday," for instance, the verb form "go" was counted as an incorrect instance. In a similar vein, the verb form "work" was deemed incorrect if the student wrote, "I work here since 2000."

c) The plural '-s':

This morpheme involves the plural '-s' and was selected due to potential L1 transfer issues. The study aimed to explore the extent to which L1 transfer influences learners' interlanguage.

### 3.3 Research instruments and materials

In this study, two main research instruments were used, a written test and an interview. These tools aimed to assess learners' performance on written tasks and gain insights into their cognitive processes.

#### 3.3.1 Written test

The written test consisted of two map-based written description tasks, each intended to provoke the application of particular target grammatical forms. There were two distinct within-task planning conditions in which the tasks were executed, pressured and unpressured. Task one, limited time (20 min) aimed to restrict participants' opportunities to devote attentional resources to grammatical repertoire. Task two, unlimited time provided ample opportunities for participants to retrieve grammatical structures. The time conditions were based on theoretical claims [8,19,15,16] related to cognitive processes, including the limitations of working memory and focus.

#### 3.3.2 Interview

The interview functioned as an auxiliary tool to collect participant insights and comprehend their thought processes while completing the task. The purpose of the interview, which was conducted right after each participant completed the two written tasks, was to extract retrospective reflections on the task completion process.

#### 3.3.3 Material

Visual cues from the Cambridge IELTS sample tests were used as material for task design. This choice aimed to facilitate language production by participants, emphasizing the expression of meaning in tasks. The study design, based on sound theoretical grounds, aligned with the IELTS description task type which provides proficiency testing in language production across diverse backgrounds. Task one, included cue words 'past' and 'present,' modified from IELTS cue words 'before' and 'future.' Task two, included cue words '1999′ and 'today,' modified from IELTS cue words 'before' and 'after. ‘This modification aimed to align the tasks with the study's objectives while maintaining the original time element of the IELTS tasks.

### 3.4 Data collection procedures

Participants physically attended the research sessions at the department of linguistics, scheduled on weekdays. The venue and schedule distribution aimed to maintain participant confidentiality and convenience. The study adhered to formal procedures, with time separately scheduled for each participant within the subject group. Participants were assigned unique codes for anonymity, used consistently across written answer sheets and interview notes. The schedule distribution and assigned codes ensured that participants could not recognize each other. Before each stage, the study's purpose was re-explained, and participants signed physical consent forms. In the following paragraphs, the process of data collection (the way in which requested participants to participate in the present study) is explained in more details.

Here is the instruction: Dear participant, you are invited to participate in a study of the ways in which the writing of Iranian second language learners of English vary under different conditions. The purpose of the study is to investigate the differences between second language writing that is done within limited time and writing that is done without time limits. Specifically, we want to understand how language learners’ approach, think about, and plan their writing under these different conditions.

If you decide to participate, you will be asked to complete two writing tasks in English which involve providing a description of pictures. One of these writing tasks will be done under time limited conditions (20 min each), while the other writing task will not have a specific time limit. Following these writing tasks, the researcher will interview you individually about your approach to the writing tasks as well as your understanding of particular English grammar rules. The interviews will be audio-recorded so that they can be transcribed later. The written tasks that participants complete will then be analyzed together with the interviews.

The total duration of the data gathering session (written tasks and interview) is expected to be about 1 h and 10 min. If you feel tired and want to take a short break between the individual writing tasks, you may certainly do so. Any information or personal details gathered in the course of the study are confidential, except as required by law. No individual participants will be identified in any publication of the results. You will be assigned a code to use when completing the written tasks and interviews, so you will not need to record your name on any of the task sheets. Participation in this study is entirely voluntary: you are not obliged to participate and if you decide to participate, you are free to withdraw at any time without having to give a reason and without consequence. I, *(participant's name*) have read and understand the information above and any questions I have asked have been answered to my satisfaction. I agree to participate in this research, knowing that I can withdraw from further participation in the research at any time without

consequence. I have been given a copy of this form to keep.

Participant's Name:

Participant's Signature: __________________________Date:

Investigator's Name:

Investigator's Signature: _____________________ ___ Date.

#### 3.4.1 Map-based picture description written task

Participants received brief instructions before starting Step one. Firstly, the participants completed a time-limited (20 min) map-based picture description task. When the allotted time ran out, the answer sheets were gathered. Participants were informed of a short break before Task two in order to avoid potential fatigue or stress effects. Finally, participants completed another picture description task without a time limit. Answer sheets were collected at the participant's discretion.

#### 3.4.2 Interview

After Stage one, participants underwent individual retrospective semi-structured interviews with pre-defined questions. Participants were provided with a note sheet to assist with some interview questions. Conducted in English and audio-recorded, with each interview lasting approximately 20 min.

Following the survey administration, a semi-structured interview was conducted with participants in the qualitative phase of the study. To this aim, 5 participants were selected for an in-depth, audio recorded, semi-structured interview. It is worth mentioning that the justification for deciding to use a semi-structured interview was that in this data collection technique, ‟the researcher uses a written list of questions as a guide, while still having the freedom to digress and probe for more information” [41].

The questions for the interview with regard to the content validity index of the items, they were reexamined by two language and two content teachers to ensure appropriateness of content and language. the researcher personally carried out a semi-structured interview with the participants. The interview sessions were conducted in the hope to bring about reliable and valid data. To this aim, the researcher initially created a friendly atmosphere to make the teachers feel comfortable. Having introduced himself, the interviewer informed the interviewees of the purpose of the interview, but avoided providing too much information about the research study in order to preclude the formation of bias in the respondents.

In a bid to gauge the reliability of the interview questions, two language experts having PhD degree in TEFL were requested to evaluate the relevance and appropriateness of the questions through a short interview session. The amount of consistency and agreement in the experts’ responses was measured and considered as the yardstick for the reliability. As pinpointed by Ref. [36]**,** the more consistent the responses, the higher is the reliability**.**

### 3.5 Data analysis

#### 3.5.1 Written task dataset

##### 3.5.1.1 Measures

It is important to note that, percentage of morpheme errors per 100 words relative to the total number of words generated in every task. In fact, the research team took into consideration morphemes in the plural "-s," past tense forms, and present tense forms.

##### 3.5.1.2 Data treatment

Two independent raters, one of whom was a native English speaker and the other non-native, checked the dataset. The two raters were university-level English and/or linguistics instructors. Inter-rater reliability was established to ensure consistency in data interpretation. Table 2 illustrates the percentage of agreement for the morphemes which were used in this study. Results showed agreement between the two raters ranging from 82.1 % to 100 % in two tasks. The researcher and the two raters conferred to settle any differences found during the data checking process.

**Table 2:** Percentage of agreement for the three types of morphemes (the plural "-s," present tense form, and past tense form).

Participant	Task 1	Task 2
1	82.1	100
2	100	86.5
3	84.6	100
4	100	100
5	100	91.7

#### 3.5.2 Interview dataset

The researchers meticulously recorded pertinent segments of the interview dataset by handwriting them down exactly. Transcription followed Mishler's [42] approach, excluding unnecessary information like redundant expressions, repeated responses, and personal details that could identify participants. Unnecessary information and unclear responses were omitted. Participants' unintentional revelation of personal information, not required for the study, was excluded. Redundant parts of participants' responses leading to the main ideas were also excluded. A strict procedure ensured objectivity in transcription, following the sequence of questions on the interview sheet. This sequential transcription facilitated a comprehensive understanding of relevant information.

## 4 Results

### 4.1 IL variability in morphological forms

**RQ1:** Under both pressured and unpressured within-task planning conditions, how much do learners exhibit patterns of IL variability in the targeted morphological forms?

#### 4.1.1 Patterns of IL variability

Three different morpheme types were taken into account in the analysis: the plural "-s," past tense forms, and present tense forms. Two distinct patterns were observed in the IL variability in morpheme errors under pressured (task one) and unpressured (task two) within-task planning conditions.

1. Decrease in Inaccuracy:

Participants P1 and P3 demonstrated a decrease in error rates from task one (pressured) to task two (unpressured). Specifically, P1 exhibited higher errors in task 1 (ratio 5.13) but significantly reduced errors in task two (ratio 1.1). Similarly, P3 displayed lower errors in task two compared to task one.

2. Increase in Inaccuracy:

Participants P2, P4, and P5 exhibited an opposite trend with higher morpheme errors in task two (unpressured) than in task one (pressured). Notably, P5 consistently demonstrated the highest error rate, with a ratio of 4.63 in task 2 compared to 2.65 in task one.

**Fig. 1:**
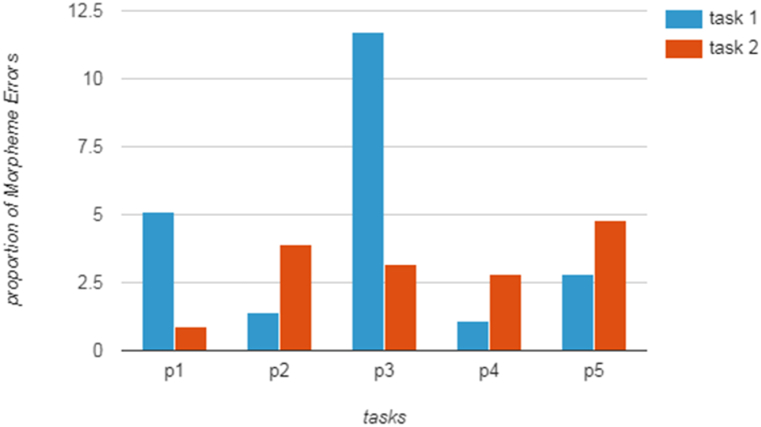
IL variability of morpheme errors in pressured (task 1) and unpressured (task 2) conditions.

The results, as illustrated in (Fig. 1), indicate that only two out of five participants (P1 and P3) displayed lower inaccuracy levels under time-unpressured conditions. This suggests that the variability in IL patterns was not consistently systematic across all participants.

The observed variability suggested individual differences in how learners respond to pressured and unpressured within-task planning conditions. The lack of consistent patterns across all participants highlighted the need for further investigation into the factors influencing IL variability in morphological forms.

#### 4.1.2 Inter-learner performance

**RQ2:** How much do students display varying degrees of accuracy in these morphological forms?

Based on Table 3, results indicated variations in accuracy levels among learners across the two tasks. The morphemes that the participants used, whether they were target-like or not, could be used to divide them into two groups.

**Table 3:** The two written tasks' improper use of morphemes.

Participant Code	Distribution of incorrect forms of three morpheme types (by %)					
	Task 1			Task 2		
	Past tense forms	Present tense forms	Plural ‘-s’	Past tense forms	Present tense forms	Plural ‘-s’
P1	0	25.3	18	0	11.3	0
P2	0	14.9	0	0	42.65	13.9
P3	73	37.2	64	19	13.2	15.1
P4	0	11.4	0	0	13.3	27.8
P5	26	11.4	16	77	19.12	43.2

##### 4.1.2.1 Group 1 (P1, P2, and P4)

Used all past tense forms for both tasks successfully using target-like forms. Showed variability in the use of the plural '-s' in both tasks. Demonstrated errors in present tense forms, with varying degrees between tasks.

##### 4.1.2.2 Group 2 (P3 and P5)

Exhibited incorrect uses in all three morpheme types in both tasks. Displayed variability in the use of these morphemes between tasks.

##### 4.1.2.3 Observations

Group one learners demonstrated success in past tense forms but exhibited variability in the use of the plural '-s' and errors in present tense forms. Group two learners consistently made errors across all three morpheme types, with variations in the degree of errors between tasks. Individual learners within each group showed differential patterns of variability in response to task planning conditions.

##### 4.1.2.4 Examples of errors

Group one: " The town's industrial area, both past and present … " (P1), "accommodations" (P2 and P4), "natural scenery remain the same" (P4), etc. Group two: " A new playground has been established," "it is built in the central" (P3), "There are a new restaurant," "beach is construct," "no more factory" (P5), etc.

Students not only showed differing degrees of improper morpheme usage but also exhibited diverse patterns of variability when transitioning from time-pressured to unpressured task planning conditions.

### 4.2 Cognitive processes: sources for IL variability

#### 4.2.1 Working memory and attention capacity

**RQ3:** How do learners' attentional resources change under pressured and unpressured within-task planning conditions?

The examination of participants' cognitive processes under pressured and unpressured conditions revealed diverse approaches. Each learner exhibited unique strategies in handling attentional resources during task completion.

##### 4.2.1.1 Pressured within-task planning conditions

**P1:** Focused more on meaning than form, emphasizing main ideas without explicit consideration of grammar rules within the limited 20-min timeframe.

*Participant Quote:* " I just prepare the key points because the first task is due in 20 min. When I write task 1, I don't give it much thought, but because task 2 requires more time, I give it more thought." (P1)

**P3:** Emphasized meaning, relying on implicit knowledge of grammar, showcasing ease in writing without intense consideration of grammatical structures.

*Participant Quote:* " I have encountered … so I can write with ease and didn't give grammar or structure a second thought." (P3)

**P2:** Leveraged unconscious grammatical knowledge during writing, acknowledging a balance between implicit and explicit rule-based knowledge.

*Participant Quote:* "Thus, I believe my grammar is rather decent. Hence, when I was writing the assignment, I did not put much effort into creating grammar or following grammatical rules. I believe that 80 % of writing should be unconscious, and the remaining 20 % should require thought." (P2)

##### 4.2.1.2 Observations

Participants, despite variations, shared a tendency to prioritize meaning over explicit grammar rules during time-pressured conditions. Different levels of reliance on implicit grammar knowledge were observed, ranging from conscious efforts to unconscious application.

Learners exhibited nuanced approaches to managing attentional resources in pressured conditions, with a common emphasis on meaning while navigating varying degrees of reliance on grammatical knowledge.

##### 4.2.1.3 Pressured and unpressured conditions: learners P4 and P5

**P4:** Centered on meaning while fusing observation and rule-based knowledge, thinking about tenses (past, present), grammatical rules (simple sentences, compound sentences) during writing.

*Participant Quote:* "Generally, when I write, I consider the tense of the sentence—past, present, etc.—as well as the grammatical rules, which include simple and compound sentences. Usually, I'll work those things out as I'm writing." (P4)

**P5:** Emphasized meaning while noticing grammatical rules before writing, allocating around 5 min before start to think about the main ideas and the appropriate verb tenses (past, present).

*Participant Quote:* "I estimate that I will start writing in 5 min. I never study for grammar rules. I just get ready for the key points. I simply consider whether I should write in the past, present, or past tense. I chose to use the past tense rather than the present tense because, for example, the first map shows an industrial area from the past." (P5)

##### 4.2.1.4 Unpressured within-task planning conditions: general observations

###### 4.2.1.4.1 Planning time utilization

Participants allocated varying durations (1–7 min) before writing to plan their task under unpressured conditions. Individual differences existed in how learners utilized this available time, emphasizing either grammar planning or focusing on main ideas.

**P1:** More time to task 2's planning and writing of grammar forms, indicating a shift from a primary focus on main ideas to incorporating grammatical considerations.

*Participant Quote:* " I just prepare the main points for the first task, which takes 20 min, and I write out the proper grammar structure for the second task." (P1)

**P4 and P5:** Shared a similar approach in both tasks, dedicating additional time in task 2 to consider grammatical structures before initiating the writing process.

*P4 Quote:* "I usually had my mind focused on the writing I was doing, so I had to be aware of tense usage, such as past and present, as well as grammatical rules regarding simple and compound sentences. Usually, I'll work those things out as I'm writing." (P4)

*P5 Quote:* "I give it 5 min or so before I start writing. I never study for grammar rules. I just get ready for the key points. I simply consider whether I should write in the past, present, or past tense. I chose to use the past tense rather than the present tense because, for example, the first map shows an industrial area from the past." (P5)

**P2:** Acknowledged awareness of required grammatical resources before writing in both tasks.

*Participant Quote:* "I know I have to compare something from the past to the present before I can begin writing. I therefore understand that when referring to something in the past, I should use the past simple, and when referring to something in the present, I should use the present perfect." (P2)

**P3:** Dedicated the initial minute to idea preparation without specific mention of grammar planning in task 2.

*Participant Quote:* "I know I have to compare something from the past to the present before I write. I therefore understand that when referring to something in the past, I should use the past simple, and when referring to something in the present, I should use the present perfect." (P3)

###### 4.2.1.4.2 Observations

Participants exhibited diverse strategies in allocating planning time and considering grammatical structures, highlighting individual preferences and shifts in attentional focus from main ideas to grammar under unpressured conditions. Variability existed in the degree of attention devoted to grammatical aspects, with some learners emphasizing these considerations more prominently than others. Learners also, demonstrated distinct patterns in utilizing available time and attentional resources before writing under unpressured conditions, showcasing individualized approaches to grammar planning and idea generation.

##### 4.2.1.5 Pressured and unpressured conditions: learners' monitoring strategies

1. P1:

###### 4.2.1.5.1 Task 2 (unpressured)

Consciously noticed grammar rules during writing. Monitored grammar before, while, and after writing.

*Participant Quote:* " I consider it both before and as I'm writing the assignment. However, after that, whenever I have time, I double-check." (P1)

2. P4:

###### 4.2.1.5.2 Task 1 (pressured)

Thought of grammar during writing.

No post-monitoring of grammar.

###### 4.2.1.5.3 Task 2 (unpressured)

Thought of grammar during writing.

Checked grammar after writing.

*Participant Quote:* "Since I am aware that I have more time, I can devote more thought to the grammar rules, including the type of structure I will employ, the tenses I will employ, and whether there are any words or vocabulary that will improve my description of the map. Since I have more time, I double-check." (P4)

3. P3 and P5:

applied implicit grammar knowledge when writing for both assignments.

Did not post-monitor grammar.

*P3 Quote:* " Similar to task 1, but without the time constraint to allow me to write for longer." (P5)

4. P2:

###### 4.2.1.5.4 Task 2 (unpressured)

Focused on meaning but noticed grammatical rules during writing.

Did not post-monitor grammar.

*Participant Quote:* "I know I have to compare something from the past to the present before I write. I therefore understand that I must use the present perfect when speaking of the present, and that when speaking of the past, I must use the past simple. But only that—I began to write." (P2)

###### 4.2.1.5.5 Post-monitoring of grammar

P1 and P4 reported post-monitoring grammar in task 2. P2, P3, and P5 did not post-monitor grammar. Variability existed in participants' strategies for monitoring grammar before, during, and after writing, demonstrating individualized approaches.

Participants exhibited diverse strategies in monitoring grammar rules during the writing process. While some learners consciously noticed and post-monitored grammar (e.g., P1, P4), others relied on implicit knowledge without post-monitoring (e.g., P2, P3, P5). The individualized nature of monitoring strategies highlights differences in cognitive processing during written task performance.

#### 4.2.2 L1 transfer and transfer of training

**RQ4:** How much does IL variability involve L1 transfer and training transfer?

##### 4.2.2.1 L1 transfer in morphological forms: evidence from interviews and translation tasks

1. P1:

###### 4.2.2.1.1 Interview response

Used to forward sentences in Iranian but now forwards sentences in English, translating only specific words from Iranian.

###### 4.2.2.1.2 Translation task

No instances of non-target-like morphological forms reported.

*Conclusion:* L1 transfer was mentioned but not evident in the translation task.

2. P4:

###### 4.2.2.1.3 Interview response

Did not switch back to Persian language for assistance.

###### 4.2.2.1.4 Translation task

Produced non-target-like morphological form ('student' instead of 'students').

*Conclusion:* L1 transfer mentioned but evident in translation task with instances of non-target-like forms.

3. P3:

###### 4.2.2.1.5 Interview response

Did not switch back to Persian language for assistance.

###### 4.2.2.1.6 Translation task

Produced non-target-like morphological form ('three year ago' instead of 'three years ago').

*Conclusion:* L1 transfer not mentioned but evident in translation task with instances of non-target-like forms.

Participants acknowledged L1 transfer in interviews, with some reporting past habits of forwarding sentences in Persian. L1 transfer manifested in translation tasks with instances of non-target-like morphological forms. The evidence suggests that L1 transfer played a role in IL variability, influencing morphological choices in English production.

###### 4.2.2.1.7 Transfer of training

No evidence from interviews indicated participants consciously applying training from ESL courses or similar experiences. Participants did not reference specific training when discussing their cognitive processes during task performance. Focus in interviews was on L1 transfer and individualized strategies rather than explicit application of training.

L1 transfer emerged as a relevant factor contributing to IL variability in morphological forms, while evidence for the transfer of training was not observed in participants' responses to the interview questions. The individualized nature of participants' cognitive processes was emphasized, with varying degrees of reliance on L1 transfer.

##### 4.2.2.2 Transfer of training: tense and plural '-s'

Tense Usage.

1. P1:

###### 4.2.2.2.1 Interview response

Could explain the usage of simple present tense but made errors in application during the tasks.

###### 4.2.2.2.2 Post-task corrections

Corrected from simple present to present perfect in one instance. Correctly used simple past tense in other instances.

2. P3:

###### 4.2.2.2.3 Interview response

Provided specific examples for the simple present tense but struggled with a general description.

###### 4.2.2.2.4 Post-task performance

Incorrectly used simple present tense in one context but correctly used it in other instances.

3. P5:

###### 4.2.2.2.5 Interview response

Made a mistake with the present perfect tense but correctly used past and simple present tenses.

###### 4.2.2.2.6 Post-task corrections

Recognized and corrected a paragraph with incorrect simple present tense to the correct simple past tense.

4. P2 and P4:

###### 4.2.2.2.7 Interview response

Correctly explained the usage of both simple past and simple present tenses.

###### 4.2.2.2.8 Post-task performance

Did not show problems with tenses in the tasks, both in interview note sheets and oral responses.

Participants demonstrated varying degrees of understanding and application of tense rules. While some made errors in task performance, post-task self-corrections were observed in a few cases. Overall, participants displayed diverse abilities in using simple past and simple present tenses, with some successfully applying the rules and others making errors.

The plural '-s' Usage.

1. P3 and P4:

###### 4.2.2.2.9 Translation task

Displayed variability in the plural '-s' usage, sometimes omitting it when required and other times using it correctly.

2. P1, P2, and P5:

###### 4.2.2.2.10 Translation task

No errors observed with the plural '-s' usage.

Participants demonstrated inconsistency in the application of the plural '-s' in the translation task. While some showed variability by occasionally omitting it, others consistently used it correctly.

The evidence suggested that participants' responses did not strongly indicate reliance on formal training in ESL courses or similar experiences for the accurate use of tense and the plural '-s'. The variability in tense usage and occasional errors in plural '-s' suggest that participants' linguistic competence in these aspects may be influenced by factors beyond formal training, such as L1 transfer and individual language learning strategies.

## 5 Discussion

The present research study P1 and P3 showed lower error rates in unpressured tasks compared to pressured tasks. Consistent with previous research [27,28,33,34], learners tend to perform better under unpressured conditions. P1 focused more on meaning than form in pressured conditions, aligning with cognitive load theory. P1, P2, and P4: Completed both tasks with at least one morpheme type using target-like forms. P3 and P5: In both tasks, the variable forms for all three morpheme types were incorrectly used. Different accuracy levels across learners indicate varied cognitive processes. Group 1 focused on target-like forms, while Group 2 displayed overall inconsistency in usage.

P1 and P4 acknowledged using Persian language for lexical items or translating sentences. Translation tasks revealed instances of non-target-like morphological forms. Participants likely affected by negative L1 transfer, contributing to IL variability. Participants demonstrated varying degrees of understanding and application. Some made errors in task performance, while post-task self-corrections were observed. Participants showed inconsistency in application, with occasional errors. Participants displayed diverse abilities, suggesting influences beyond formal training. Cognitive processes underpin IL variability. Unpressured conditions allow for better performance. L1 transfer likely contributes to IL variability. Transfer of training influence is inconclusive, with diverse participant responses. The explanatory approach integrates cognitive processes with task performance, providing a comprehensive understanding of IL variability. Working memory, attention capacity, L1 transfer, and transfer of training collectively contribute to learners' morphological variability under different planning conditions.

P1 focused more on meaning than form under time pressure. Errors reflect the reliance on implicit grammatical knowledge due to cognitive constraints. P1 engaged in conscious monitoring of grammar rules throughout task 2. Utilized unlimited time for online planning and post-writing checks. Resulted in higher accuracy due to careful planning [21,2,43]. Explicit knowledge automatized to implicit knowledge over time. Post-task self-correction demonstrated reliance on explicit knowledge. P3's case suggests the automatization of explicit knowledge into implicit. Evidence from self-correction indicates the dynamic nature of learners' cognitive processes. P1: Explicit online planning in unpressured conditions led to higher accuracy. P3: Implicit grammatical knowledge became automatized, reducing errors in task two.

Cognitive processes indicate dynamic shifts between explicit and implicit knowledge. Transfer of explicit knowledge contributes to improved accuracy over time. The discussion highlights the dynamic nature of learners' cognitive processes, demonstrating how explicit knowledge transforms into implicit knowledge. P1's conscious planning and monitoring under unpressured conditions contrast with P3's automated use of grammar rules. These findings underscore the nuanced interplay of cognitive factors in shaping interlanguage variability. P2, P4, and P5 displayed higher inaccuracies in unpressured conditions. In contrast to expectations and prior studies, working memory limitations didn't explain this pattern. Learners did not use morpheme forms randomly; psycholinguistic processes were involved. Support for Ellis' [44] suggestion that free variation occurs when IL is unaffected by psycholinguistic factors.

Learners exhibited differential levels of success in each morpheme type. Results align with [28,37] findings on individual variations. Two distinct patterns observed in learners' performance. Limited working memory alone can't explain variability; other psycholinguistic factors contribute. Findings don't align with studies showing consistency under task conditions. Learners exhibited unique patterns not captured by previous research. Cognitive processes dynamically influence task performance. Learners navigate psycholinguistic complexities differently. Varied cognitive processes contribute to interlanguage variability. The discussion emphasizes the dynamic and multifaceted nature of cognitive processes in shaping interlanguage variability. The findings provide nuanced insights into the interplay of psycholinguistic factors, working memory, and attention capacity in learners' performance. Acknowledging individual differences and tailoring teaching strategies accordingly can contribute to more effective language instruction.

Working memory and attention capacity alone don't fully explain IL variability. L1 transfer and transfer of training likely contribute to mixed patterns. Learners demonstrated varying degrees of success in recognizing and correcting errors. Multiple cognitive factors at play, beyond just working memory limitations. Working memory, attention, L1 transfer, and transfer of training may jointly affect IL production. Errors may stem from a combination of cognitive sources, supporting the notion of multifactorial influences. Participants failed to notice and correct all non-target-like forms. Challenges in detecting and addressing errors suggest the involvement of factors beyond attention and working memory constraints. This discussion underscores the intricate nature of IL production, emphasizing the need for a comprehensive approach to error analysis in language teaching. Recognizing the joint influence of multiple cognitive factors offers valuable insights for designing targeted interventions and fostering a nuanced understanding of language learners' challenges.

Learners occasionally omitted 3SG-s and/or the plural '-s' in English IL production. Participants demonstrated features of their native language in English production. Learners acknowledged switching back to Persian for English writing (e.g., P4). Errors in the translation task (e.g., P3) further indicated L1 transfer effects. Consistent with prior studies [28,34] on the role of L1 transfer in IL variability. Supports Long's [33] assertion that mother tongue interference contributes to non-target-like elements. Participants occasionally used the tense incorrectly in various contexts. Some learners were able to self-correct tense errors (e.g., P1 correcting present perfect tense). Three learners used the plural '-s' in inappropriate contexts (e.g., 'accommodations'). Reflects systematic variability in interlanguage production [44]. Responses to interviews did not strongly support transfer of training as a factor. Unique errors in unpressured conditions suggest factors beyond limited working memory. bolsters the arguments made by James [32] and Johnson [45]‍ regarding insufficient grammatical knowledge. Complex cognitive processes influence learners' behavior in producing interlanguage. Further investigation required to determine the extent of transfer of training's contribution to IL variability.

## 6 Conclusions

This study shed light on the cognitive processes and interlanguage behaviors of Iranian ESL students. The students displayed two divergent patterns of variability in the plural "-s," past tense forms, and present tense forms. Under relaxed circumstances, some students made fewer mistakes while others made more mistakes. Under both time-pressured (task one) and unpressured (task two) conditions, they displayed varying degrees of accuracy in each morpheme type. learners used varying use of target-like forms among learners across morpheme types. Allocation of attentional resources to meaning and/or form varied among individual learners. They manifested different ways of engaging with tasks under pressured and unpressured planning conditions. L1 transfer identified as a possible influential factor in learners' task performance. Evidence from interviews suggested the negative influence of L1 on morpheme forms.

The study enriches evidence about variations in morphological forms among L2 learners, especially in the context of Iranian English language learning. Task planning conditions (timed vs. untimed) can impact IL variability, providing insights into how learners adapt to different conditions. Regularly administering tasks under time pressure conditions can help learners adapt and improve their ability to pay conscious attention to both meaning and grammatical forms. Familiarity with time constraints can aid learners in real-life situations, such as formal examinations. Teachers should recognize that IL variability results from a combination of cognitive factors, not a singular one. Awareness of potential joint effects of working memory, attention, and L1 transfer is crucial for understanding learners' language production. L1 transfer is identified as a contributing factor to IL variability. Teachers should consider the potential impact of learners' native language on their English language production. The study suggests an approach to elicit potential psycholinguistic factors through retrospective interviews, revealing the complexity of learners' mental processes.

Teachers can gain insights into how learners' psycholinguistic processes manifest under different time conditions. Pedagogical interventions should consider the observed sources of IL variability, such as working memory, attention, and L1 transfer. Providing timely feedback and obtaining learners' retrospections can be valuable in addressing specific cognitive processes leading to errors. While there's no conclusive evidence on transfer of training, teachers should be aware of instances where learners use language features inappropriately. Further exploration and consideration of this aspect can contribute to a comprehensive understanding of IL variability.

Despite providing valuable pedagogical insights, the study has certain limitations that necessitate further investigation: A relatively small sample of Iranian English language learners participated in the study, limiting the generalizability of the results to broader populations with different linguistic backgrounds. The exploratory nature of the case study posed limitations on generalization. The findings may not be universally applicable to learners with diverse language backgrounds. While attempts were made to uncover cognitive processes through interviews, the evidence supporting theoretical grounds, such as the engagement of mother tongue interference and transfer of training, remains limited. Learners may not provide a comprehensive account of all mental processes, and Certain transfer processes can be applied both consciously and unconsciously. In order to explain IL variation, the study concentrated on particular cognitive constructs (working memory, attention, L1 transfer, and transfer of training). Other psycholinguistic processes that could contribute to IL variability were not examined. The use of a picture task description may be less cognitively demanding than other task types, potentially impacting the interpretation of time pressure effects. The study did not explore the optimal time limit for tasks, and variations in time constraints could yield different findings. The study ignored other factors like learner proficiency level, which may contribute to IL variability among learners at different proficiency levels, and instead concentrated mostly on within-task planning conditions.

The study suggested several directions for future research to enhance understanding and contribute to the field of interlanguage (IL) variability: Further investigation is recommended to gain a more nuanced understanding of individual learners' IL, utilizing learners' retrospection. This involves exploring additional psycholinguistic processes beyond the scope of the current study to comprehend the complexity of learners' mental processes under different task planning conditions. Future studies should take into account both external and internal variables, such as learners' past task-type knowledge, proficiency levels, and other essential components. Variations in IL variability and inter-learner differences in performance could be caused by these factors. Future studies are encouraged to examine tense and verb forms simultaneously, given the observed variability in learners' responses in written tasks. Understanding how learners use tenses inappropriately, leading to incorrect verb forms, will contribute to a more comprehensive analysis.

The scope of targeted linguistic forms should be expanded to include other grammatical structures beyond the morphological forms examined in the current study. Exploring areas such as the use of articles may provide insights into inter-learner differential success across a wider range of grammatical features. Future research should aim to include a larger and more diverse population, exploring IL variability in English as a second language (ESL) learners and learners of English as a foreign language. This comparative approach will help understand how learning environments and linguistic backgrounds influence task performance. Comparative studies involving learners from different linguistic backgrounds are recommended. Exploring IL variability among learners of culturally diversified backgrounds can provide insights into how grammatical errors may vary across different L1 source languages. Future research is encouraged to incorporate the above points, especially at the PhD level. This method is anticipated to produce far-reaching pedagogical implications in a wider educational context in addition to theoretical knowledge related to second language acquisition.

## Ethics statement

The study was approved by the ethical commission of Yazd University, Iran (case number 2023–6188). All participants provided written informed consent for the use of their data for scientific research purposes.

Financial support and sponsorship: our study did not receive specific funding or sponsorship.

## Data availability statement

Data associated with the study has not been deposited into a publicly available repository. Data are available from the corresponding author on reasonable request.

## Funding

This study dose not receive specific funding or sponsorship.

## CRediT authorship contribution statement

**Forough Amirjalili:** Writing – review & editing, Writing – original draft, Visualization, Validation, Supervision, Software, Resources, Project administration, Methodology, Investigation, Funding acquisition, Formal analysis, Data curation, Conceptualization. **Masoud Neysani:** Writing – review & editing, Writing – original draft, Methodology, Investigation. **Ahmadreza Nikbakht:** Writing – review & editing, Writing – original draft, Investigation.

## Declaration of competing interest

The authors declare that they have no known competing financial interests or personal relationships that could have appeared to influence the work reported in this paper.
