# Supplementary material for: Unraveling interlanguage variability: A psycholinguistic exploration of morphological forms in Iranian ESL learners' written performance
Source: Heliyon. 2024 May 24;10(11):e31582. doi: 10.1016/j.heliyon.2024.e31582 (PMC11153087; doi:10.1016/j.heliyon.2024.e31582)
Supplement: Multimedia component 1 [file mmc1.docx]

**Appendices**

**Appendix A**

**INTERVIEW QUESTIONS**

Welcome to the **participant with code**………to the Interview. This interview is designed to

collect data for the purpose of the present research study. Before the interview starts, I will share

the following information with you:

First, the interview is audio-recorded.

Second, I will provide you with a note sheet to give your answers where questions require.

Third, some questions will ask you to look at your written task performance to give your answers.

All of the information you provide in this interview will be used for academic purposes only.

There are no right or wrong answers to the questions so please feel free to answer the questions

as honestly as possible.

**PART 1**. (Questions related to the participants’ conscious monitoring of within-task

performance under time conditions)

1. When you were completing Task 1 with the **time limit** (20 minutes):

1.1. How did you approach the activity in the task? (How did you plan your task? What did

you do within 20 minutes in order to be able to complete your task? In what way were you

able to complete the task within 20 minutes? How do you use 20 minutes effectively in order to

be able complete the task?)

1.2. How much time did you plan/ prepare your task 1, like plan/ prepare grammar rules

before your writing within 20 minutes?)? -How did you apply/ use grammar rules in your

writing? i.e., you apply/use the grammar rules unconsciously/ automatically or did you

think of grammar rules when you write your task? If so when did you think of the

grammar rules, i.e., before, during or after you write your task during this 20 minute?

1. When you were completing Task 2 **with no time limit**:

2.1. How did you approach the activity? (How did you plan your task? What did you do within

20 minutes in order to be able to complete your task? In what way were you able to complete

the task with at least 120 words with no time limit? How do you use the unlimited time in

order to be able complete the task?)

2.2. How much time did you plan/ prepare your task 2, like plan/ prepare grammar rules

before your writing with no time limit?) - How did you apply/ use grammar rules in your

writing? , i.e. you apply/use the grammar rules unconsciously/ automatically or did you think

of grammar rules when you write your task? If so when did you think of the grammar rules, i.e.

before, during or after you write your task during this 20 minutes?

3. Comparing the writing task with a time limit and the writing task with no time limit, what

differences (if any) did you feel when completing them?

**PART 2** (Questions related to internal factors which have influenced the participants’ task

performance)

4. When you wrote an English sentence, in what condition do/ did ever you plan/ think a

Persian sentence in order to write an English sentence? Could you translate this Persian

sentence into English?

از سال 2000 تا 2005، مسعود و مریم همیشه دانش آموزان ممتاز مدرسه بودند زیرا آنها فعالانه درگیر یادگیری و سایر فعالیت‌ها هستند.

Participant’s translation version:………………………………

مسعود اغلب در طول تعطیلات تابستانی یک دوره زبان انگلیسی می گذراند اما دوستانش اغلب تعطیلات خود را با انجام فعالیت های اجتماعی می گذرانند.

Participant’s translation version: …………………

من یک دیکشنری انگلیسی دارم که دوستم سه سال پیش بهم داده است، اما من دو ماه بعد گمش کردم.

Participant’s translation version: ………………..…………

در آن مدرسه شاگردان خوب زیاد هستند و یکی از آنها مریم است.

Participant’s translation version: …………………

5. Specific questions regarding past tense and present tense:

5.1. Can you write the structure of past tense? How would you explain the structure of the past

tense?

5.2. How would you explain the use of the past tense in English?

5.3. Can you write the structure of present tense? How would you explain the structure of the

present tense?

5.4. How would you explain the use of the present tense in English?

6. Where did you learn about these English language forms (past tense and present tense) (e.g.

from teachers? from books?, etc…)?

7. Can you complete the sentences with the verbs in brackets?

a) He (complete)………… his master degree in 2015 and (get)…………. a job at a

company one year after that.

b) Some of her friends usually (join)………….. group discussions but Maryam (tend)……...

to work independently.

c) She (drop)……….… a course in management two years ago and she (study)………..

another course two months after that.

d) Masoud always (be)………….. active in groupwork activities in class but not all his

friends (be)…………… like him.

8. (Look at your performance on two tasks with time limit and with no time limit):

8.1. Could you write out any verbs that were used in the wrong present/ past tense(s) in

Task 1? Why do you think the error(s) occurred when you were completing this task within

20 minutes?/ Why were not you aware of the error(s) when you were completing task 1?

Can you change the error(s) in the correct form?

8.2. Could you write out any verbs that were used in the wrong present/past tense(s) in Task

2? Why do you think the error(s) occurred when you were completing this task with no time

limit? Why were not you aware of the error(s) when you were completing task 2? Can you

change the error(s) in the correct form?

Thank you for your participation!

**INTERVIEW NOTE SHEET**

Participant code:……….

**PART 2**.

4. Translation

از سال 2000 تا 2005، مسعود و مریم همیشه دانش آموزان ممتاز مدرسه بودند زیرا آنها فعالانه درگیر یادگیری و سایر فعالیت‌ها هستند.

………………………………………………………………………………

مسعود اغلب در طول تعطیلات تابستانی یک دوره زبان انگلیسی می گذراند اما دوستانش اغلب تعطیلات خود را با انجام فعالیت های اجتماعی می گذرانند.

……………………………………………………………………………

من یک دیکشنری انگلیسی دارم که دوستم سه سال پیش بهم داده است، اما من دو ماه بعد گمش کردم.

………………………………………………………………………………

در آن مدرسه شاگردان خوب زیاد هستند و یکی از آنها مریم است.

………………………………………………………………………………

5. Grammatical structure:

5.1. Past tense………………………………………….

5.3. Present tense: …………………………………….

7. Sentence completion

a) He (complete)……..…… his master degree in 2015 and (get)…………. a job at a

company one year after that.

b) Some of her friends usually (join)………….. group discussions but Maryam (tend)……...

to work independently.

c) She (drop)……….… a course in management two years ago and she (study)………..

another course two months after that.

d) Masoud always (be)………….. active in groupwork activities in class but not all his

friends (be)…………… like him.

8. Realization of the tenses used

8.1. Present tense form:

8.2. Past tense form:

Thank you for your participation!

**Appendix B**

**MAP-BASED PICTURE DESCRIPTION FOR WRITTEN TASKS**

**Task 1**

The maps below show an industrial area in the town of Norbiton in the past and at the

present.

Summarize the information by selecting and reporting the main features, and make comparisons

where relevant.

You are given 20 minutes to complete this task.

You should write at least 150 words.

Norbiton industrial area in the past

**Norbiton industrial area at the present**

Task 2

The two maps below show the construction of some tourist facilities in an island in 1999

and today.

Summarize the information by selecting and reporting the main features, and make comparisons

where relevant.

You can spend as much time as you wish to complete this task.

You should write with at least 150 words.

In 1999

\
